# Minor hallucinations as an early marker to differentiate dementia with Lewy bodies from Alzheimer's disease

**DOI:** 10.1177/13872877261468798

**Published:** 2026-07-29

**Authors:** Marina Ruiz Piñero, Natalia Pérez Carmona, Belen Piñol Ferrer, Ángel Pérez Sempere, Inmaculada Abellán Miralles

**Affiliations:** 1 Memory Unit, Neurology Department, 146244 Hospital San Vicente del Raspeig, Sant Vicent del Raspeig, Spain; 2 Neurology Department, 16802 Dr Balmis General University Hospital, Alicante, Spain; 3 Department of Clinical Medicine, 16753 Miguel Hernandez University, Elche, Spain; 4 Alicante Institute for Health and Biomedical Research (ISABIAL), Alicante, Spain

**Keywords:** Alzheimer's disease, dementia with Lewy bodies, differential diagnosis, early diagnosis, hallucinations, pareidolia

## Abstract

**Background:**

Early differentiation between Alzheimer's disease (AD) and dementia with Lewy bodies (DLB) remains clinically challenging, particularly in the initial stages, when cognitive profiles largely overlap. Accurate diagnosis is increasingly relevant given disease-specific therapeutic implications.

**Objective:**

To compare clinical, neuropsychological, and perceptual features of early-stage AD and DLB, with a particular focus on minor hallucinations (MH), and to assess their diagnostic utility in differentiating both conditions.

**Methods:**

This prospective, cross-sectional study included 121 patients with probable AD (n = 60) or DLB (n = 61), all in mild stages (GDS 3–4) and supported by disease-specific biomarkers. Participants underwent standardized clinical assessment, comprehensive neuropsychological evaluation, structured evaluation of psychotic symptoms, and perceptual testing, including the Pareidolia Test. Group comparisons and ROC analyses were performed to assess discriminative accuracy.

**Results:**

Global cognition, attention, executive functions, language, and visuospatial abilities largely overlapped between groups. Episodic memory impairment was significantly more severe in AD across all recall measures. In contrast, DLB patients showed a higher prevalence of core clinical features and a markedly higher frequency of MH (54.1% versus 13.3% in AD). Pareidolic responses were significantly more frequent in DLB and showed the highest discriminative accuracy among perceptual measures. The presence of MH demonstrated a high positive predictive value for DLB.

**Conclusions:**

In early disease stages, MH and susceptibility to perceptual distortions differentiate DLB from AD more effectively than standard cognitive testing. Systematic assessment of these phenomena may improve early diagnostic accuracy in routine clinical practice.

## Introduction

Differentiating Alzheimer's disease (AD) from dementia with Lewy bodies (DLB) in the early stages of cognitive decline remains a major challenge in clinical neurology. Both conditions may initially present with mild cognitive impairment or behavioral changes, but they differ in prognosis, therapeutic response, and management. This distinction has become more relevant with the emergence of disease-modifying anti-amyloid therapies, which are specific to AD. Early and accurate differential diagnosis is therefore critical.

Visual hallucinations are a well-established hallmark of DLB and are included among its core diagnostic criteria.^1^ Recurrent, well-formed visual hallucinations are reported in approximately 60–80% of patients with DLB over the course of the disease.^2^ In contrast, hallucinations are uncommon in the early stages of AD; large memory-clinic cohorts report prevalences as low as 4–5% in patients with probable AD during early disease phases.^3^

Beyond fully developed visual hallucinations, increasing attention has been directed toward *minor visual phenomena*, a spectrum of subtle perceptual disturbances that include minor hallucinations (MH) (presence hallucinations and passage hallucinations), visual illusions, and pareidolias. These phenomena are frequent in Lewy body disease and have been proposed as potential early manifestations of the disease. However, it remains unclear whether they precede complex visual hallucinations or represent parallel perceptual alterations.^4^

Systematic reviews have reported a high prevalence of minor visual phenomena in Lewy body disease, including Parkinson's disease and DLB, with wide variability across studies.^4^ In contrast, MH have been far less studied in AD, and available data suggest that they are uncommon in early stages, although estimates vary depending on diagnostic criteria and assessment methods.^5–9^

Importantly, no previous studies have systematically examined MH in very early, biomarker-defined AD and DLB populations using structured perceptual assessments. Most existing studies have relied on clinical diagnoses, included heterogeneous disease stages, or lacked a direct comparison between the two conditions. Consequently, the diagnostic value of MH for differentiating AD from DLB at the earliest stages—when clinical decision-making is most challenging—remains unknown.

The aim of this study was to compare the clinical, neuropsychological, and perceptual profiles of patients with early-stage DLB and AD, with a particular focus on MH. Specifically, we sought to characterize the prevalence and phenomenology of MH in both conditions, evaluate their capacity to discriminate between DLB and AD, and examine their potential usefulness as a supportive clinical tool for differential diagnosis in routine clinical practice.

## Methods

### Study design and setting

This was a prospective, observational, cross-sectional study conducted at a single specialized dementia unit. Participants were consecutively recruited from the Dementia Unit of San Vicente del Raspeig Hospital, Spain. The study was designed to characterize perceptual disturbances and associated clinical and neuropsychological features in the early stages of AD and DLB, and to compare these features between both diagnostic groups.

All participants underwent a standardized diagnostic work-up according to the unit's clinical protocol, which includes neurological examination, neuroimaging, biomarker assessment, structured clinical questionnaires and a comprehensive neuropsychological evaluation administered by a trained examiner.

### Participants

Patients were eligible for inclusion if they met current clinical criteria for probable AD or probable DLB and if the diagnosis was supported by biomarker evidence.

AD was diagnosed according to the 2018 National Institute on Aging–Alzheimer's Association (NIA-AA) criteria,^10^ requiring confirmation through amyloid positron emission tomography (PET) or cerebrospinal fluid (CSF) biomarkers. DLB was diagnosed in accordance with the 2017/2020 consensus criteria of the Dementia with Lewy Bodies Consortium.^1,11^ All patients with DLB were required to present at least one positive indicative biomarker, including abnormal dopamine transporter imaging (DaTSCAN), reduced cardiac uptake on metaiodobenzylguanidine (MIBG) scintigraphy, or polysomnographic confirmation of REM sleep behavior disorder (RBD).

Only individuals in the mild stages of cognitive impairment were included, defined as Global Deterioration Scale (GDS) stages 3 or 4. GDS 3 was considered consistent with mild cognitive impairment/prodromal disease, with preserved independence in both basic and instrumental activities of daily living. GDS 4 corresponded to mild dementia, characterized by impairment in instrumental activities of daily living while preserving autonomy in basic activities. Exclusion criteria comprised more advanced dementia (GDS ≥5), the presence of other neurological disorders potentially affecting cognition, significant visual or auditory impairment that could interfere with assessment, and a history of alcohol or substance abuse. Patients fulfilling criteria for Parkinson's disease dementia were excluded according to the temporal criteria established by the DLB Consortium (“1-year rule”). In addition, cases with high clinical suspicion of relevant mixed pathology or biomarker evidence suggestive of co-pathology were not included in the study cohort. To minimize potential pharmacological confounding effects on perceptual symptoms and motor findings, patients receiving medications potentially influencing these domains—including neuroleptics, dopaminergic therapy, memantine, cholinesterase inhibitors, or opioids—were also excluded.

### Clinical assessment

Sociodemographic data collected included age, sex and years of formal education. Clinical variables comprised family history of dementia, vascular risk factors, psychiatric history, sensory deficits and current or previous use of psychotropic medication. Dementia-related clinical characteristics were systematically recorded, including diagnostic category, age at diagnosis, duration of symptoms and GDS score. The presence of core clinical features of DLB was specifically documented.

Parkinsonian motor signs were assessed using a five-item version of the Unified Parkinson's Disease Rating Scale (UPDRS-5) as proposed in the Assessment Toolkit for Dementia with Lewy Bodies developed in the DIAMOND Lewy study.^12^ REM sleep behavior disorder assessment was based on clinical history obtained from patients and caregivers and supported by the questionnaire included in the DIAMOND Lewy assessment toolkit.^12^ Cognitive fluctuations and hallucinations—including MH and other perceptual disturbances—were evaluated using validated questionnaires and structured clinical interviews.

### Neuropsychological assessment

All participants completed a brief cognitive screening battery consisting of the Mini-Mental State Examination (MMSE), the *Test de Alteración de la Memoria* (T@M), copying of intersecting pentagons, the clock drawing test and semantic and phonemic verbal fluency tasks.

In addition, a comprehensive neuropsychological battery was administered by an examiner blinded to the clinical diagnosis. Attention and executive function were assessed using the Trail Making Test parts A and B, Digit Span, and semantic and phonemic verbal fluency tasks. Memory and learning were evaluated with the Free and Cued Selective Reminding Test (FCSRT). Working memory was assessed using Digit Span backwards. Language abilities were explored through the Boston Naming Test and comprehension and repetition subtests from the Barcelona Test. Orientation was evaluated using the Barcelona orientation subscore, and praxis was assessed through tasks of symbolic gesture, imitation of postures and motor sequencing.

Visuoconstructive abilities were examined using the geometric figure copying task from the Barcelona Test. Visuoperceptual processing was specifically assessed through selected subtests of the Visual Object and Space Perception Battery, including number location, silhouettes and object decision.

Visual perceptual susceptibility to illusions was additionally assessed using the Pareidolia Test,^13^ which evaluates the tendency to perceive meaningful images in ambiguous visual stimuli. The number of pareidolic responses, omissions and total errors were recorded.

### Ethics

The study was approved by the institutional ethics committee (Ref. CEIm: PI2021-122; ISABIAL: 2021-0301). Written informed consent was obtained from all participants or their legal representatives in accordance with the Declaration of Helsinki.

### Statistical analyses

Statistical analyses were conducted using R software, version 4.4.2 R Foundation for Statistical Computing, Vienna, Austria; http://www.r-project.org) A statistical significance level of 0.05 was set, and all tests were two-tailed. The normality of continuous variables was assessed using the Kolmogorov–Smirnov test with Lilliefors correction.

Since most variables did not follow a normal distribution, results were reported as median (p25; p75) and compared between diagnostic groups (AD versus DLB) using the Mann–Whitney U test. Categorical variables were summarized as n (%) and compared using either the Fisher's exact test or the chi-squared test, as appropriate.

To evaluate the discriminative ability of the different measures, the area under the receiver operating characteristic curve (AUC) was calculated using the pROC package. This analysis was used to determine the optimal cut-off points for the different scores of the pareidolia test and the UPDRS, both for the total score and for its subscales. In addition, performance metrics such as sensitivity and specificity were obtained, along with 95% confidence intervals (95% CI) for the AUC, which were estimated using bootstrap resampling with 2000 iterations.

Further, stratified subanalyses were performed for participants with and without MH to explore potential differences in cut-off point performance.

## Results

A total of 121 participants were included in the study, with 60 diagnosed with AD and 61 with DLB. Patients with DLB were slightly older, with a median age of 75 years (IQR 70–78) compared to 71 years (IQR 68–74) in the AD group (p = 0.001). The median time from symptom onset to diagnosis was also longer in the DLB group, at 36 months (IQR 18–48) versus 24 months (IQR 18–36) in AD (p = 0.037). The proportion of participants in stages GDS 3 and GDS 4 was similar between groups, with the majority classified as stage 3 in both DLB (62.3%) and AD (65.0%).

Regarding clinical characteristics, patients with DLB had a lower prevalence of dyslipidemia compared with AD (50.8% versus 69.5%, p = 0.042), but a higher prevalence of depression (42.6% versus 18.3%, p = 0.005), and were more frequently treated with antidepressants (44.3% versus 25.4%, p = 0.036) and benzodiazepines (26.2% versus 10.0%, p = 0.032). Family history of dementia was less common in DLB (36.1%) than in AD (66.7%, p = 0.001). Other factors, including sex distribution, hypertension, diabetes, obesity, sleep apnea, smoking, alcohol use, anxiety, hypnotic use, and education level, did not differ significantly between groups (Table 1).

**Table 1.** Sociodemographic and clinical characteristics.

	AD (n = 60)	DLB (n = 61)	**p**
Sex, % (women)	66.7% (40)	60.7% (37)	ns
Age, median (IQR)	71 (68–74)	75 (70–78)	**0.001**
Time to diagnosis (months)	24 (18–36)	36 (18–48)	**0.037**
GDS = 3	65.0% (39)	62.3% (38)	ns
GDS = 4	35.0% (21)	37.7% (23)	ns
Education level, % (n)			
≤Primary education	60.0% (36)	62.3% (38)	ns
Secondary education	25.0% (15)	27.9% (17)	ns
University education	15.0% (9)	9.8% (6)	ns
Hypertension, % (n)	46.7% (28)	63.9% (39)	ns
Diabetes mellitus, % (n)	23.3% (14)	18.0% (11)	ns
Dyslipidemia, % (n)	69.5% (41)	50.8% (31)	**0.042**
Obesity, % (n)	3.3% (2)	11.5% (7)	ns
Obstructive sleep apnea, % (n)	11.7% (7)	6.6% (4)	ns
Smoking, % (n)	16.7% (10)	8.2% (5)	ns
Alcohol consumption, % (n)	1.7% (1)	8.2% (5)	ns
Anxiety, % (n)	15.0% (9)	19.7% (12)	ns
Depression, % (n)	18.3% (11)	42.6% (26)	**0.005**
Antidepressant use, % (n)	25.4% (15)	44.3% (27)	**0.036**
Benzodiazepine use, % (n)	10.0% (6)	26.2% (16)	**0.032**
Hypnotic use, % (n)	3.3% (2)	4.9% (3)	ns
Family history, % (n)	66.7% (40)	36.1% (22)	**0.001**

Neuropsychological performance was largely comparable between groups across most cognitive domains. No consistent between-group differences were observed in attention or executive functions (Trail Making Test A/B, digit span, serial subtractions, abstraction) or in language measures (naming, repetition, fluency/grammar, information content). Visuoperceptual performance was also broadly similar on VOSP indices and subtests, and constructional abilities did not clearly differentiate the groups at this early stage, with the exception of the number of angles in the pentagon drawing, which was lower in participants with DLB (p = 0.014). Global cognitive performance, as measured by the MMSE, was likewise comparable.

In contrast, memory measures showed a clear dissociation between groups. Participants with AD performed significantly worse on verbal episodic memory on the FCSRT (total free recall, total recall, delayed free recall, and delayed total recall; all p < 0.001), as well as on the T@M test (total score, free recall, and cued recall; all p < 0.001) (Table 2). Overall, both groups showed relative preservation of non-mnestic domains, whereas memory impairment was more severe in the AD group and was not improved by cueing, compared with the DLB group.

**Table 2.** Neuropsychological performance.

COGNITIVE SCREENING WITH BRIEF TESTS							
Domain / Test	Total Median (IQR)	n	AD Median (IQR)	n	DLB Median (IQR)	n	p
MMSE (median, IQR)							
Total score	26.0 (22.0; 28.0)	121	25.0 (21.8; 27.0)	60	27.0 (22.0; 28.0)	61	0.139
Pentagon test (median, IQR)							
Total score	13.0 (11.0; 13.0)	121	13.0 (12.0; 13.0)	60	13.0 (10.0; 13.0)	61	0.185
T@M (median, IQR)							
Total score	32.0 (28.0; 38.0)	121	29.5 (24.0; 35.0)	60	35.0 (31.0; 40.0)	61	**<0** **.** **001**
Immediate memory	8.0 (7.0; 9.0)	121	8.0 (6.8; 9.0)	60	8.0 (8.0; 9.0)	61	0.153
Temporal orientation	4.0 (3.0; 5.0)	121	4.0 (3.0; 5.0)	60	4.0 (3.0; 5.0)	61	0.098
Semantic memory	13.0 (11.0; 14.0)	121	13.0 (10.8; 14.0)	60	13.0 (11.0; 14.0)	61	0.985
Free recall	2.0 (0.0; 4.0)	121	1.5 (0.0; 2.2)	60	4.0 (1.0; 5.0)	61	**<0**.**001**
Cued recall	6.0 (4.0; 8.0)	121	4.0 (2.8; 7.0)	60	7.0 (6.0; 9.0)	61	**<0**.**001**
Pentagon test (median, IQR)							
Total score	13.0 (11.0; 13.0)	121	13.0 (12.0; 13.0)	60	13.0 (10.0; 13.0)	61	0.166
Number of angles	4.0 (3.0; 4.0)	121	4.0 (4.0; 4.0)	60	4.0 (3.0; 4.0)	61	**0**.**014**
Distance or intersection	4.0 (3.0; 4.0)	121	4.0 (4.0; 4.0)	60	4.0 (3.0; 4.0)	61	0.898
Closure or opening	2.0 (2.0; 2.0)	121	2.0 (2.0; 2.0)	60	2.0 (2.0; 2.0)	61	0.075
Rotation	2.0 (2.0; 2.0)	121	2.0 (2.0; 2.0)	60	2.0 (2.0; 2.0)	61	0.166
Closing-in	1.0 (1.0; 1.0)	121	1.0 (1.0; 1.0)	60	1.0 (1.0; 1.0)	61	0.317
Clock drawing test (median, IQR)							
Total score	8.0 (5.6; 10.0)	121	8.0 (5.0; 10.0)	60	8.0 (6.0; 10.0)	61	0.443
Circle	2.0 (2.0; 2.0)	121	2.0 (2.0; 2.0)	60	2.0 (2.0; 2.0)	61	0.580
Numbers	4.0 (2.0; 4.0)	121	4.0 (2.0; 4.0)	60	4.0 (2.0; 4.0)	61	0.517
Hands	3.0 (1.0; 4.0)	121	3.0 (1.0; 4.0)	60	3.0 (1.0; 4.0)	61	0.579
NEUROPSYCHOLOGICAL STUDY							
Attention and cognitive functions							
Trail Making Test - Part A	7.0 (4.0; 9.0)	117	7.0 (5.0; 9.0)	56	6.0 (3.0; 8.0)	61	0.248
Trail Making Test - Part B	7.0 (5.0; 10.0)	89	6.0 (5.0; 10.0)	45	7.0 (5.0; 11.8)	44	0.383
Digits: forward	10.0 (9.0; 13.0)	113	10.5 (9.0; 13.0)	56	10.0 (9.0; 12.0)	57	0.220
Digits: backward	10.0 (7.0; 11.0)	113	10.0 (8.0; 12.0)	56	10.0 (7.0; 11.0)	57	0.244
Serial subtraction: forward order	18.0 (18.0; 18.0)	113	18.0 (18.0; 18.0)	56	18.0 (18.0; 18.0)	57	0.532
Serial subtraction (forward) - time	8.0 (6.0; 18.0)	113	18.0 (8.0; 18.0)	56	8.0 (4.0; 18.0)	57	0.115
Serial subtraction: backward order	6.0 (5.0; 18.0)	113	6.0 (6.0; 18.0)	56	6.0 (4.0; 18.0)	57	0.390
Serial subtraction (backward) - time	8.0 (6.0; 10.0)	112	8.0 (6.0; 11.0)	56	8.0 (6.0; 10.0)	56	0.432
Arithmetic problems	8.0 (6.5; 10.0)	111	8.0 (7.0; 10.0)	56	8.0 (6.0; 11.0)	55	0.903
Arithmetic problems - time	8.0 (6.0; 9.0)	111	8.0 (6.0; 9.0)	56	8.0 (6.0; 9.5)	55	0.814
Abstraction: similarities	10.0 (8.0; 11.0)	110	10.0 (8.0; 11.0)	56	10.0 (8.0; 11.0)	54	0.819
Language							
Boston Naming Test	9.0 (8.0; 11.0)	118	9.0 (8.0; 11.0)	57	9.0 (8.0; 11.0)	61	1.000
Fluency and grammar	18.0 (18.0; 18.0)	113	18.0 (18.0; 18.0)	56	18.0 (18.0; 18.0)	57	0.495
Information content	18.0 (18.0; 18.0)	113	18.0 (18.0; 18.0)	56	18.0 (18.0; 18.0)	57	0.580
Repetition: nonwords	8.0 (5.0; 18.0)	110	8.0 (5.5; 18.0)	55	8.0 (5.0; 18.0)	55	0.075
Repetition: words	18.0 (18.0; 18.0)	120	18.0 (18.0; 18.0)	59	18.0 (18.0; 18.0)	61	0.793
Category fluency: animals	7.0 (5.0; 9.0)	120	7.0 (5.0; 10.0)	59	7.0 (5.0; 8.0)	61	0.270
Comprehension: following commands	10.0 (8.0; 18.0)	112	9.5 (6.8; 18.0)	56	14.0 (8.0; 18.0)	56	0.260
Memory and learning							
FCSRT Free Recall trial 1	7.0 (5.0; 10.0)	112	7.0 (5.0; 9.0)	55	8.0 (6.0; 10.0)	57	0.080
FCSRT Total Free Recall	5.0 (3.0; 7.0)	112	4.0 (2.0; 5.5)	55	6.0 (4.0; 7.0)	57	**<0**.**001**
FCSRT Total Recall	4.0 (3.0; 7.0)	112	3.0 (2.0; 5.0)	55	6.0 (3.0; 8.0)	57	**<0**.**001**
FCSRT Delayed Free Recall	3.0 (2.0; 6.0)	112	2.0 (2.0; 4.0)	55	5.0 (3.0; 7.0)	57	**<0**.**001**
FCSRT Delayed Total Recall	4.0 (3.0; 7.0)	112	3.0 (3.0; 4.0)	55	5.0 (4.0; 8.0)	57	**<0**.**001**
Orientation							
Person	18.0 (3.0; 18.0)	113	18.0 (3.0; 18.0)	56	18.0 (3.0; 18.0)	57	0.307
Place	18.0 (3.0; 18.0)	113	18.0 (3.0; 18.0)	56	18.0 (3.0; 18.0)	57	0.928
Time	4.0 (2.0; 18.0)	113	4.0 (2.0; 18.0)	56	6.0 (2.0; 18.0)	57	0.213
Visuoperceptual function							
VOSP - Incomplete Letters	18.0 (15.0; 19.0)	115	18.0 (16.0; 19.0)	59	18.0 (14.8; 19.2)	56	0.910
VOSP - Silhouettes	15.0 (11.0; 20.0)	121	15.0 (10.8; 19.2)	60	16.0 (12.0; 20.0)	61	0.672
VOSP - Object Decision	9.0 (8.0; 11.0)	67	9.0 (8.0; 11.0)	39	8.0 (7.0; 11.0)	28	0.551
VOSP - Progressive Silhouettes	8.5 (7.0; 10.0)	62	8.0 (7.0; 10.0)	37	9.0 (7.0; 10.0)	25	0.628
VOSP - Dot Counting	10.0 (9.0; 10.0)	121	10.0 (9.0; 10.0)	60	10.0 (9.0; 10.0)	61	0.645
VOSP - Position Discrimination	19.0 (18.0; 20.0)	119	19.0 (18.0; 20.0)	58	19.0 (18.0; 20.0)	61	0.592
VOSP - Number Location	9.0 (5.2; 16.0)	66	9.0 (5.2; 16.8)	38	9.0 (6.5; 12.2)	28	0.891
VOSP - Cube Analysis	8.0 (5.0; 9.0)	119	8.0 (6.0; 9.0)	58	7.0 (4.0; 9.0)	61	0.207
Praxis							
Symbolic gesture	18.0 (6.0; 18.0)	114	18.0 (6.0; 18.0)	57	18.0 (18.0; 18.0)	57	0.225
Imitation of postures: bilateral	18.0 (5.0; 18.0)	112	18.0 (5.0; 18.0)	57	9.0 (5.0; 18.0)	55	0.530
Posture sequences: right	7.0 (5.0; 9.0)	113	8.0 (5.0; 9.0)	56	6.0 (5.0; 9.0)	57	0.518
Constructional praxis (copy)	8.0 (6.0; 11.0)	111	9.0 (7.0; 12.0)	55	7.0 (6.0; 9.2)	56	0.121

### Pareidolia test

Performance on the Pareidolia Test differed significantly between diagnostic groups (Table 3): although median total error scores were similar between groups, patients with DLB showed a wider distribution of errors, resulting in a significant group difference (p = 0.001). The number of pareidolic responses was significantly higher in the DLB group than in the AD group (median [IQR]: 2.0 [0.0–7.0] versus 0.0 [0.0–1.0], respectively; p = 0.008), indicating a greater tendency toward illusory visual perceptions in DLB. Omissions were infrequent overall; although median omission scores were zero in both groups, the DLB group showed greater variability, resulting in a statistically significant difference between groups (p = 0.035). Therefore, perceptual misinterpretation measures favored DLB, consistent with greater vulnerability to visuoperceptual distortions.

**Table 3.** Pareidolia test.

Domain / test	Total median (IQR)	n	AD median (IQR)	n	DLB median (IQR)	n	p
N° mistakes	39.0 (36.0; 40.0)	121	39.0 (38.0; 40.0)	60	38.0 (32.0; 40.0)	61	0.001
N° pareidolias	1.0 (0.0; 3.0)	121	0.0 (0.0; 1.0)	60	2.0 (0.0; 7.0)	61	0.008
N° omission	0.0 (0.0; 0.0)	121	0.0 (0.0; 0.0)	60	0.0 (0.0; 1.0)	61	0.035

**Figure 1.**
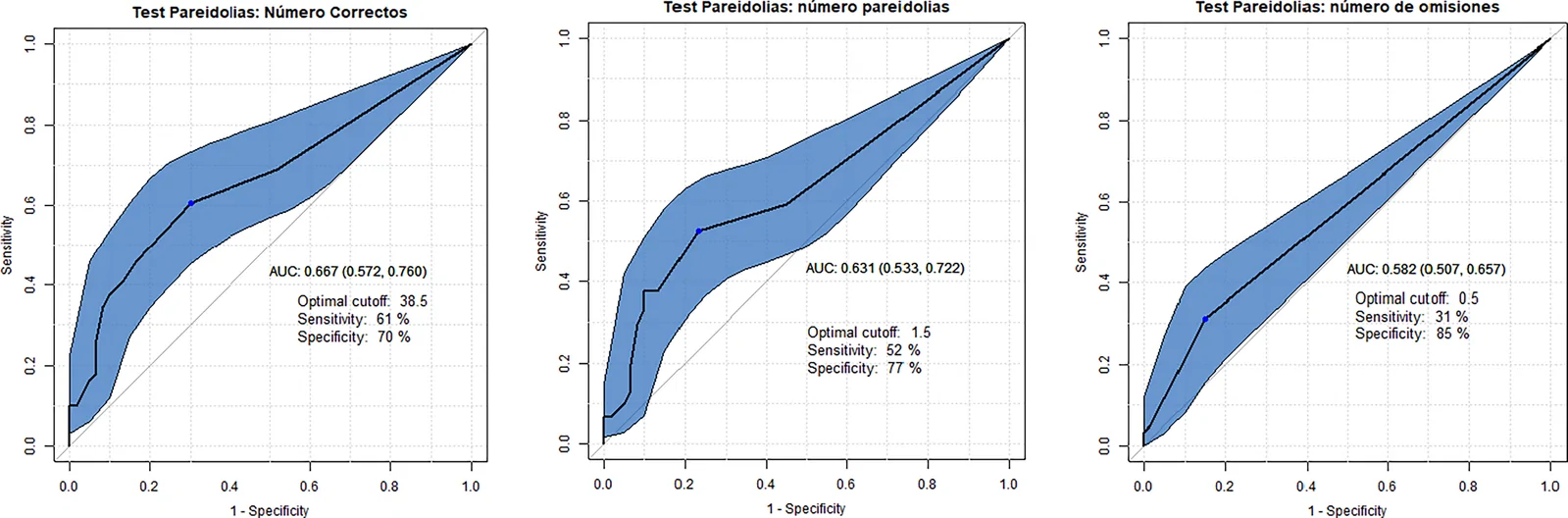
Pareidolia test. ROC analyses.

ROC analyses (Figure 1) showed that the number of pareidolic responses had the highest discriminative accuracy for differentiating DLB from AD, yielding a higher area under the curve (AUC) than total errors and omissions. Higher numbers of pareidolic responses were more frequently observed in the DLB group, whereas low or absent pareidolic responses were more common in AD.

Overall, these findings indicate that, in early disease stages, standard cognitive and language testing has limited discriminative value between AD and DLB, whereas episodic memory (worse in AD) and perceptual error propensity (more marked in DLB) provide the most informative contrasts.

### Core symptoms

As expected, participants diagnosed with DLB showed a markedly higher prevalence of core clinical features compared with those with AD, even from early stages (Table 4). REM sleep behavior disorder, cognitive fluctuations, and visual hallucinations (VH) were all significantly more frequent in the DLB group whereas they were rare in the AD group (55.0% versus 10.0%; 29.5% versus 3.3% and 27.9% versus 1.7%, respectively; all p < 0.001). In addition, overall parkinsonian motor burden, assessed by the UPDRS-5 score, was substantially greater in DLB than in AD. This difference was mainly driven by higher scores for bradykinesia, rigidity, and reduced facial expression in DLB, while tremor did not differ between groups (predominantly rigid-akinetic parkinsonian phenotype).

**Table 4.** Description of DLB core symptoms in participants of the study, overall and by diagnosis.

	Total (n = 121)	AD (n = 60)	DLB (n = 61)	p
RSBD	32.5% (39)	10.0% (6)	55.0% (33)	<0.001
Fluctuations, % (n)	16.5% (20)	3.3% (2)	29.5% (18)	<0.001
Visual hallucinations, % (n)	14.9% (18)	1.7% (1)	27.9% (17)	<0.001
UPDRS-5, median (RI)				
Total score	2.0 (1.0; 4.0)	1.0 (0.0; 1.2)	4.0 (3.0; 6.0)	<0.001
Rest tremor	0.0 (0.0; 0.0)	0.0 (0.0; 0.0)	0.0 (0.0; 0.0)	0.480
Kinetic tremor	0.0 (0.0; 0.0)	0.0 (0.0; 0.0)	0.0 (0.0; 0.0)	0.042
Facial expression	0.0 (0.0; 0.0)	0.0 (0.0; 0.0)	0.0 (0.0; 1.0)	<0.001
Bradykinesia	1.0 (0.0; 2.0)	0.0 (0.0; 0.0)	2.0 (1.0; 2.0)	<0.001
Rigidity	1.0 (0.0; 2.0)	0.0 (0.0; 1.0)	2.0 (1.0; 2.0)	<0.001

### Other psychotic symptoms

Psychotic symptoms differed significantly between diagnostic groups (Table 5). MH were reported by 54.1% of participants with DLB compared with 13.3% of those with AD (p < 0.001), with presence and passage hallucinations—either isolated or combined—being substantially more prevalent in DLB. In contrast, illusions were infrequent and did not differ between groups (p = 0.119). Auditory hallucinations were infrequent overall and occurred exclusively in the DLB group (8.2% versus 0.0%), although this difference did not reach statistical significance (p = 0.057). Illusions, delusions, and delusional misidentification were rare and did not differ significantly between groups.

**Table 5.** Description of other neuropsychiatric symptoms in participants of the shadow study, overall and by diagnosis.

	Total (n = 121)	AD (n = 60)	DLB (n = 61)	p
Illusions, % (n)	3.3% (4)	0.0% (0)	6.6% (4)	0.119
Presence hallucinations, % (n)	21.5% (26)	5.0% (3)	37.7% (23)	<0.001
Passage hallucinations, % (n)	27.3% (33)	11.7% (7)	42.6% (26)	<0.001
Minor hallucinations (type), % (n)				<0.001
Any	33.9% (41)	13.3% (8)	54.1% (33)	
Both	14.9% (18)	3.3% (2)	26.2% (16)	
Presence only	6.6% (8)	1.7% (1)	11.5% (7)	
Passage only	12.4% (15)	8.3% (5)	16.4% (10)	
MH + Visual	10,7% (13)	0.0% (0)	21.3% (13)	
Auditory hallucinations, % (n)	4.1% (5)	0.0% (0)	8.2% (5)	0.057
Somatosensory hallucinations, % (n)	1.6% (2)	0.0% (0)	3.3% (2)	0.160
Delusions, % (n)	4.1% (5)	1.7% (1)	6.6% (4)	0.365
Delusional misidentification, % (n)	0.8% (1)	0.0% (0)	1.6% (1)	1.000

In the DLB group, MH were highly prevalent and emerged as the second most frequent clinical manifestation (54.1%), closely following REM sleep behavior disorder (55.0%). Notably, MH were more common than other core clinical features, including cognitive fluctuations (29.5%) and VH (27.9%). This distribution highlights the prominent presence of MH in patients with DLB within this cohort.

From another perspective, when patients were stratified according to the presence of MH, 41 of 121 participants (33.9%) reported at least one type of MH. Of these, 8 (19.5%) had AD and 33 (80.5%) had DLB. The presence of MH yielded a positive predictive value of 0.80 for DLB, indicating that 80% of patients with MH were diagnosed with DLB rather than AD. Sensitivity and specificity were 0.54 and 0.87, respectively, while the negative predictive value was 0.65 (Table 6).

**Table 6.** Indicators according to the presence of MH.

		Diagnosis		
		AD	DLB	Total
Minor hallucinations	Yes	8	33	41
	No	52	28	80
	Total	60	61	121

From a descriptive perspective, MH were characterized as low-intensity phenomena, generally associated with minimal emotional impact and preserved insight. Their reported frequency was low, typically occurring several times per month. Presence and passage hallucinations were most commonly related to people—sometimes familiar individuals—and less frequently to animals.

Complex VH were predominantly described as involving significant persons, most often close family members, frequently deceased, and occasionally childhood versions of living relatives. These hallucinations were generally brief, occurred mainly in the home environment, and in some cases had a hypnagogic character. In this group, insight was more variable, while emotional impact was usually low to moderate.

Regarding other hallucinatory modalities, auditory hallucinations were typically described as hearing one's name being called or perceiving sounds such as a door opening or a doorbell ringing. Olfactory hallucinations involved the perception of person-specific odors (e.g., perfume, tobacco, sweat). Somatosensory hallucinations were mainly reported as sensations of something on the skin (insects crawling).

The most frequently described delusional symptoms included ideas of theft, persecution, and delusional misidentification.

### Subanalysis: minor hallucination yes/no

A subanalysis was conducted stratifying participants according to the presence or absence of MH within each diagnostic group (AD and DLB). Sociodemographic, clinical, motor, psychiatric, and neuropsychological variables were compared between subgroups.

No statistically significant differences were observed between patients with and without MH in either diagnostic group across sociodemographic or clinical characteristics, motor severity as assessed by the Unified Parkinson's Disease Rating Scale (UPDRS), cognitive screening measures, or performance on the standard neuropsychological assessment.

Regarding psychotic symptoms, VH differed significantly according to the presence of MH (p = 0.044), being more frequent among patients with DLB and MH compared with those without. No other psychotic symptoms showed statistically significant differences according to the presence of MH in either diagnostic group.

## Discussion

In this cohort of patients in early disease stages (GDS 3–4), cognitive profiles of AD and DLB largely overlapped, while perceptual phenomena, particularly minor hallucinations and pareidolic responses, provided the most informative features for differentiating both conditions. Standard neuropsychological measures of attention, executive function, language, visuospatial abilities, and global cognition did not consistently differentiate between the two diagnostic groups. The main exception was episodic memory: patients with AD performed significantly worse than those with DLB on all FCSRT and T@M memory measures, affecting both free and cued recall. This pattern is consistent with a genuine storage/consolidation deficit associated with hippocampal involvement and confirms episodic memory impairment as the most robust early cognitive feature of AD compared with DLB.^14^

The cognitive profile proposed for prodromal DLB, based on a recent systematic review and meta-analysis, is characterized by poorer performance compared with healthy controls in attention/processing speed, executive functions, and visuospatial abilities.^15^ When compared with AD at the mild cognitive impairment stage, individuals with prodromal DLB show worse performance in attention and processing speed, but relatively better performance in memory.^16^ However, our findings did not fully replicate this cognitive pattern. This discrepancy may reflect differences in cohort composition, biomarker-based diagnostic confirmation, and the focus on early disease stages in the present study: visuospatial deficits classically associated with DLB may be less evident in these early symptomatic stages, particularly when assessed using conventional neuropsychological measures.

In contrast to the limited discriminative value of standard cognitive measures, perceptual phenomena appeared to provide additional information for differential diagnosis. Patients with DLB showed higher rates of pareidolic responses, greater prevalence of MH and a higher frequency of VH. Pareidolic responses demonstrated the highest discriminative accuracy in ROC analyses, suggesting that perceptual alterations may be useful in distinguishing early DLB from early AD. These findings are in line with a recent systematic review on minor visual phenomena in Lewy body disease, which reported that patients with DLB exhibit more pareidolic responses than patients with AD and healthy controls. That review also highlighted the potential utility of scene and noise pareidolia tests, particularly face-based versions, in differentiating DLB from AD, a distinction that has been observed even at the prodromal stage, with higher rates of pareidolias reported in MCI-DLB compared with MCI-AD.^4^ These observations support the notion that vulnerability to visuoperceptual distortions represents an early and relevant feature of DLB.

One of the most notable findings of the present study is the high prevalence of MH in DLB, affecting more than half of patients and occurring more frequently than fully formed visual hallucinations. In contrast, MH were uncommon in AD.

The literature on MH in AD is limited and heterogeneous. Reported prevalence varies widely depending on disease stage and diagnostic definition. In one of the few studies focusing explicitly on MH in AD^6^ reported a prevalence of 21.1% in patients with AD, compared with 12.8% in amnestic mild cognitive impairment and 3% in cognitively healthy controls. However, in that study the AD group included patients across different stages of dementia, and the amnestic MCI group was not biomarker-defined, limiting comparability with cohorts restricted to early disease stages. More recently,^17^ in a large comparative study of probable DLB and probable AD excluding severe dementia, found MH in 50.6% of DLB patients and 27.7% of AD patients, although participants were not specifically limited to mild stages of disease. Likewise, in a cross-sectional study of newly diagnosed patients within three years of symptom onset,^5^ found that hallucinations were markedly more frequent in DLB. Taken together, these studies with our findings, suggest that MH are considerably more common in DLB than in AD, particularly in early stages, and reinforce their potential value as early clinical markers distinguishing DLB from AD.

In Parkinson's disease, MH are common, occur early, and may precede both motor symptoms and fully formed visual hallucinations. Prospective studies in newly diagnosed, drug-naïve patients have reported MH in up to 40% of cases^18^. Their high prevalence and early occurrence led to their inclusion in the NINDS–NIMH diagnostic criteria for Parkinson's disease psychosis, and longitudinal data indicate that MH predict the subsequent development of structured hallucinations, independently of dopaminergic treatment.^19^ This body of evidence supports that MH represent an early important manifestation of Lewy body–related neurodegeneration.

From a diagnostic perspective, the presence of MH showed a high positive predictive value for DLB, indicating that approximately eight out of ten patients presenting with these symptoms were ultimately diagnosed with DLB. Sensitivity was moderate (0.54), whereas specificity was high (0.87), and the negative predictive value was 0.65. These findings are clinically relevant because MH are easy to assess with targeted questioning but are frequently under-recognized in clinical practice.

The co-occurrence of increased pareidolic responses and the high prevalence of MH and VH in DLB suggests a continuum of perceptual disturbances. Pareidolias may represent early or mild expressions of the same mechanisms that later give rise to hallucinations. This interpretation is reinforced by the subanalysis: MH were not associated with greater cognitive, motor, or psychiatric severity, but were specifically linked to a higher frequency of VH. This indicates that MH are not merely markers of advanced disease, but reflect a distinct perceptual vulnerability within DLB.

A recent review by Pagonabarraga et al. proposes that these phenomena arise from the interaction between impaired bottom-up sensory processing and dysfunctional attentional control networks. Early posterior cortical degeneration may degrade sensory input, while failure of attentional control mechanisms allows internally generated representations to intrude into conscious perception.^19^

The present findings have direct implications for early clinical assessment. Systematic evaluation of MH and the incorporation of simple perceptual tests, such as the Pareidolia Test, may provide diagnostically relevant information in cases where conventional neuropsychological assessment fails to distinguish between AD and DLB. Integrating these elements into routine clinical practice could facilitate earlier and more accurate identification of DLB and help reduce the diagnostic delay commonly observed in these patients.

Given their high prevalence, early emergence, and strong positive predictive value, MH may warrant greater consideration in future refinements of diagnostic criteria for DLB. Longitudinal studies are needed to clarify their prognostic value and to better define their temporal relationship with the development of fully formed VH and other core clinical features.

Several limitations should be acknowledged. First, neuropathological confirmation was not available; however, all participants fulfilled criteria for probable diagnoses supported by disease-specific biomarkers, which substantially reduces diagnostic uncertainty. Although cases with high clinical suspicion or biomarker evidence suggestive of relevant mixed pathology were excluded, concomitant co-pathology cannot be completely excluded in vivo. Second, the single-center design may limit generalizability, although it ensured methodological consistency and a homogeneous clinical assessment. Third, the cross-sectional nature of the study precludes longitudinal evaluation of the evolution of hallucinations over time; nonetheless, this design is appropriate for the exploratory characterization of early phenomenological differences. Finally, hallucinations are inherently subjective phenomena and may be underreported or variably captured depending on patient insight and caregiver input; to mitigate this, information was systematically obtained from both sources whenever possible.

### Conclusion

In early stages of disease, AD and DLB show substantial cognitive overlap, with episodic memory impairment remaining the key marker of AD. In contrast, susceptibility to perceptual distortions, manifesting as pareidolias and minor hallucinations, emerges as the most informative and distinctive feature of early DLB. Systematic assessment of minor hallucinations and the incorporation of simple perceptual tests such as the Pareidolia Test may provide clinically useful information for the early differentiation between AD and DLB.
